# Quercetin Affects Erythropoiesis and Heart Mitochondrial Function in Mice

**DOI:** 10.1155/2015/836301

**Published:** 2015-05-28

**Authors:** Lina M. Ruiz, Celia Salazar, Erik Jensen, Paula A. Ruiz, William Tiznado, Rodrigo A. Quintanilla, Marlen Barreto, Alvaro A. Elorza

**Affiliations:** ^1^Center of Biomedical Research, Faculty of Health Sciences, Universidad Autónoma de Chile, Ricardo Morales 3369, 8910132 Santiago, Chile; ^2^Center for Biomedical Research, Faculty of Biological Sciences and Faculty of Medicine, Universidad Andres Bello, República 217, 8370146 Santiago, Chile; ^3^iEng Solutions Ltd., London N12 0DR, UK; ^4^Department of Chemical Sciences, Faculty of Exact Sciences, Universidad Andres Bello, República 275, 8370146 Santiago, Chile; ^5^Millennium Institute of Immunology and Immunotherapy, Santiago, Chile

## Abstract

Quercetin, a dietary flavonoid used as a food supplement, showed powerful antioxidant effects in different cellular models. However, recent *in vitro* and *in vivo* studies in mammals have suggested a prooxidant effect of quercetin and described an interaction with mitochondria causing an increase in O_2_^∙−^ production, a decrease in ATP levels, and impairment of respiratory chain in liver tissue. Therefore, because of its dual actions, we studied the effect of quercetin *in vivo* to analyze heart mitochondrial function and erythropoiesis. Mice were injected with 50 mg/kg of quercetin for 15 days. Treatment with quercetin decreased body weight, serum insulin, and ceruloplasmin levels as compared with untreated mice. Along with an impaired antioxidant capacity in plasma, quercetin-treated mice showed a significant delay on erythropoiesis progression. Heart mitochondrial function was also impaired displaying more protein oxidation and less activity for IV, respectively, than no-treated mice. In addition, a significant reduction in the protein expression levels of Mitofusin 2 and Voltage-Dependent Anion Carrier was observed. All these results suggest that quercetin affects erythropoiesis and mitochondrial function and then its potential use as a dietary supplement should be reexamined.

## 1. Introduction

The generation of reactive oxygen species (ROS) due to normal cell metabolism and the accumulative damage they cause to DNA, proteins, and lipid membranes have been associated with the development of many acquired diseases and aging. Thus, antioxidant therapies, especially through the intake of nutraceutical pills as food supplement, have become popular in our communities. However,* in vivo* studies of the antioxidant properties of dietary flavonoids have shown some paradoxical effects on human health [1] making important to investigate further and deeper the mechanism of action of these supplements.

Quercetin is one of the most abundant dietary flavonoids, with the highest antioxidant capability [2, 3], modulating the expression of different antioxidant enzymes such a catalase and superoxide dismutase, and increasing the intracellular levels of glutathione [4–6]. Furthermore, multiple and diverse functions have been ascribed to quercetin such as an antihypertensive [7], anticoagulant [8], antiatherogenic [7], antibacterial [9], and antiproliferative [10]. In last years, conflicting biological effects of quercetin have been reported, which might be related to its metabolites, its dose, and the cellular redox state [11–14]. During ROS scavenging process, quercetin gets oxidized, and it further reacts with glutathione and the protein thiol groups, leading to consumption of glutathione, an increase in cytosolic calcium concentration and lactate dehydrogenase leakage [11].

Quercetin may interact directly with mitochondrial membranes [15, 16] affecting its fluidity. This has also been associated with mitochondrial dysfunction through the inhibition of respiratory chain or uncoupling [15, 16]. In addition, quercetin affects mitochondrial calcium regulation, increases O_2_^∙−^ production, and induces the opening of mitochondrial transition pore (mPTP) in HCT116 cells [17]. The above studies suggest quercetin could promote oxidation affecting mitochondrial function, raising concerns about the validity of quercetin as an antioxidant [17].

At molecular level, quercetin is potent iron chelator, which is shown to alter the expression of proteins involved in iron absorption affecting iron homeostasis [18, 19]. Iron is an essential element in each of the four mitochondrial electron transfer complexes, either as Fe/S cluster's or heme's component [20]. Iron deficiency leads to altered cell metabolism [20] and anemia [21, 22]. Red blood cell development is the most iron requiring process for oxygen transport as well as the most active cell generator system involving both proliferation and differentiation from hematopoietic stem cells, which are also dependent on mitochondrial metabolism [21, 23]. Additionally, iron deficiency-induced anemia can have deleterious effects on heart [24], and cardiomyopathy development is related with mitochondrial dysfunction due to ROS excess [25, 26]. Both erythropoiesis and the cardiac tissue are then suitable and attractive targets for quercetin mechanistic studies.

Our working hypothesis is that quercetin interferes with mitochondrial function exacerbating mitochondrial ROS generation and altering the physiology of tissues highly dependent on iron metabolism and mitochondrial function such as the erythroid and cardiac tissue. We are interested in addressing the* in vivo* prooxidant effect of quercetin on mitochondrial function in these tissues.

Adaptive responses of mitochondria to maintain cell's bioenergetics capacity under stressful conditions involve the remodeling of mitochondrial respiratory complexes to build up or down supramolecular structures called supercomplexes; and the mitochondrial fusion and fission events called mitochondrial dynamics. The former will allow a better substrate channeling to preclude extreme production of ROS from the normal respiratory chain function [27–29]. The latter will control energy expenditure and metabolic reprogramming [30–33]. Upregulation of Mitofusin 2 (MFN2) protein, involved in mitochondrial fusion and then with elongated mitochondria, has been associated with a protective role against apoptosis, hypoxia, and ROS [32, 34, 35], as well as with higher oxidative capacity by regulating in part the respiratory complex proteins expression [35–38]. MFN2 expression is regulated in turn by the peroxisome proliferator activated receptor-gamma coactivator-1*α* (PGC-1*α*) under a variety of conditions characterized by energy expenditure [39]. PGC-1*α* is cofactor that participates in the regulation of mitochondrial biogenesis and activation of peroxisome proliferator activated receptor-*γ* (PPAR *γ*) pathway [40, 41].

Our results showed that quercetin clearly affected mitochondrial function in mice. Interestingly, quercetin decreased erythropoiesis and reduced the expression levels of mitochondrial proteins that control mitochondrial dynamics. These results proposed that the antioxidant properties of quercetin need to be reevaluated given their widespread use [42].

## 2. Materials and Methods 

### 2.1. Animals and Experimental Design

Male C57BL/6 mice (12 months of age) were daily administered intraperitoneally (i.p.) with 50 mg/kg quercetin (Cat # Q4951, Sigma) or with vehicle (5% DMSO) and PBS for control animals, during 15 days. Animals were daily checked for weight and health conditions. Right after the treatments, animals were submitted to different tests indicated below and then sacrificed for heart and bone marrow dissections. I.p. injections of quercetin in rodents have been reported previously in [43–47], and the dose of 50 mg/kg of quercetin has been reported in [47–50].

### 2.2. Strength Test

To evaluate strength, resistance, and exercise abilities, mice performed the Kondziela's test and a weightlifting test. Briefly, Kondziela's inverted screen is a test for muscle strength using all four limbs, in which each mouse was placed in the center of a screen, and then rotated, to an inverted position with the mouse's head declining first; the mice falling time was recorded. The performance of each mouse in the inverted screen was scored as follows: Falling between 1–10 sec = 1; 11–25 sec = 2; 26–60 sec = 3; over 60 sec = 4 [51]. On the other hand, the weightlifting is a test for forelimbs muscle strength. The weightlifting assessed the ability to raise seven weights ranging from 20 g to 98 g (20, 33, 46, 59, 72, 85, and 98 g). First, the mouse was allowed to raise the lightest weight (20 g) for 3 sec and up to 3 times. After a 10 sec rest in between each lift, the second and third raises were performed to move onto the next heaviest weight. The trial finishes when the mouse fails to lift or hold the weight after three attempts, recording the maximum time/weight achieved. The score calculations was made according to [51] and normalized by the body weight.

### 2.3. Bone Marrow Analyses

Bone marrow was isolated from both legs and immunostained with the antibodies phycoerythrin- (PE-) conjugated anti-TER119 and fluorescein isothiocyanate- (FITC-) conjugated anti-CD71 according to [30, 52]. Progression of erythropoiesis was then assessed by flow cytometry [30].

### 2.4. Total Antioxidant Capacity

Antioxidant capacity was evaluated using OxiSelect Total Antioxidant Capacity (TAC) Assay Kit (Cat # STA-360, Cell Biolabs, Inc.) according to manufacturer's instructions. Results are expressed as “*μ*M Copper Reducing Equivalents” and compared with control and quercetin-treated samples.

### 2.5. Insulin Determination

For the quantitative determination of insulin in mouse plasma was used the Mouse Insulin ELISA kit (Cat # 80-INSMS-E01, APLCO) according with the manufacturer's instructions [53].

### 2.6. Western Blot

Mitochondrial proteins were prepared from fresh isolated mitochondria solubilized in 20 mM HEPES, 2 mM EDTA, 0.5% Triton-X100, 150 mM NaCl, 1 mM PMSF, and a HALT protease cocktail inhibitor [30]. These were then buffered and fractionated in 8% Bis-Tris polyacrylamide gel with MOPS and a 5 mM sodium bisulfite running buffer before being transferred onto a 0.2 *μ*m PVDF membrane with NUPAGE transfer buffer in the semidry apparatus. The antibodies PGC1-*α*+*β* (Cat # ab72230, Abcam), MFN2 (Cat # ab50843, Abcam), VDAC1/Porin (Cat # ab15895, Abcam), ceruloplasmin (Cat # ab8813, Abcam), and *β*-actin (Cat # ab8227, Abcam) were used according to a previous study [30, 32]. Detection of carbonyl groups introduced into proteins by oxidative stress was performed with the OxyBlot Protein Oxidation Detection Kit (Cat # S7150 Millipore) according to [30, 32]. The quantity of ceruloplasmin was evaluated in plasma samples, mixing 5 *μ*L of plasma, 5 *μ*L of sample buffer, and 15 *μ*L of H_2_O. The sample was boiled at 95°C for 10 min and centrifuged and run in a protein electrophoresis and blotted in PVDF membrane for ceruloplasmin immunodetection [30].

### 2.7. Blue Native Polyacrylamide Gel Electrophoresis (BN-PAGE)

Mitochondria were isolated by differential centrifugation. Mitochondrial proteins were solubilized with the NativePAGE Sample Prep Kit, and 80 *μ*g per well were loaded onto a 3–12% polyacrylamide gradient NativePAGE Novex Bis-Tris Gel (Invitrogen, Carlsbad, CA) [30].

### 2.8. In-Gel Activity Assay (IGA)

Complex I* in-gel* activity (CI-IGA) was detected by incubating BN-PAGE gels right after electrophoresis in 100 mM Tris-HCl, pH 7.4, with 1 mg/mL nitro blue tetrazolium and 0.14 mM NADH at room temperature for 60 min in the dark with gentle rocking [54]. Complex IV* in-gel* activity (CIV-IGA) was detected by incubating the gel with 0.1% (w/v) 3,3′-diaminobenzidine, 0.1% (w/v) cytochrome c, and 24 units/mL catalase in 1 mM Tris-HCl, pH 7.4, at 37°C for 6 h in the dark with gentle rocking [28, 55]. Complex II* in-gel* activity (CII-IGA) was detected by incubating the gel in 5 mM Tris-HCl, pH 7.4, with 20 mM sodium succinate, 0.2 mM phenazine methosulfate and 2.5 mg/mL nitro blue tetrazolium [30, 56].

### 2.9. Statistics

Statistical analyses were performed with Origin Pro8 with a significance level set at *P* ≤ 0.05. Unpaired Student's *t*-test was used when comparing 2 average values.

## 3. Results

### 3.1. Effects of Quercetin on Metabolism and Strength Support in Mice

**Figure 1:**
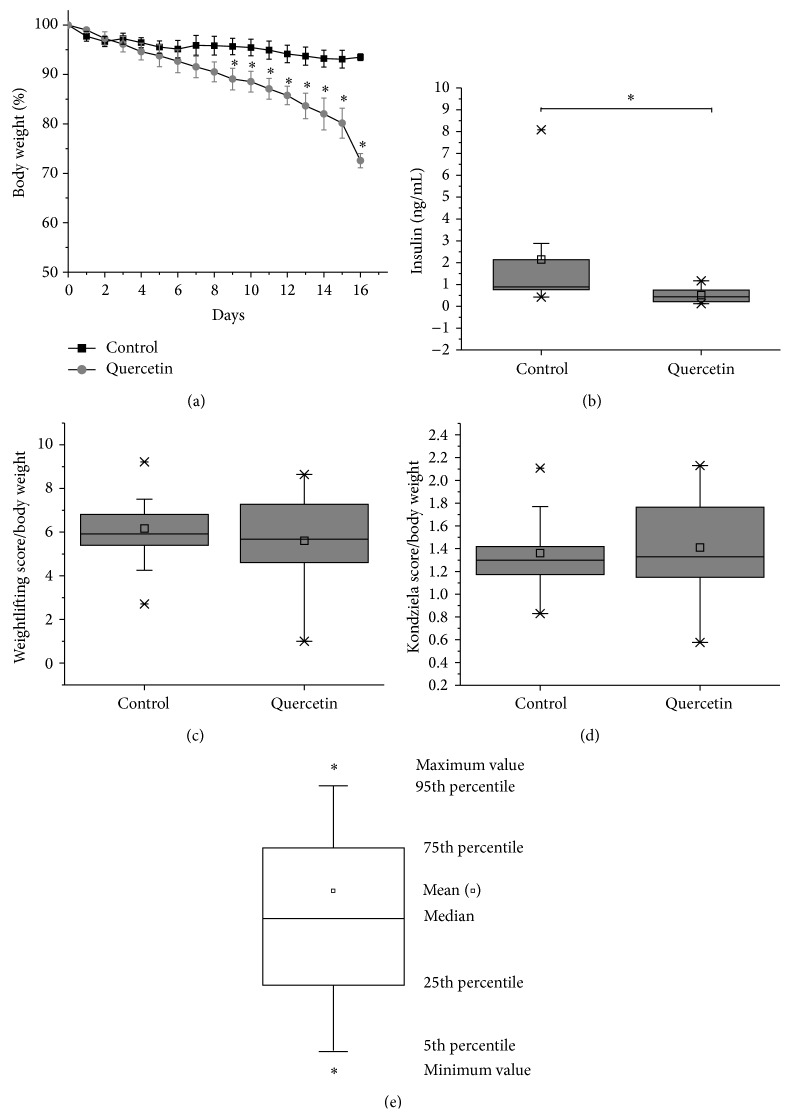
Effects of quercetin treatment on weight and plasma insulin levels in mice. Quercetin modulates mice metabolism. (a) Mice were treated with quercetin (grey circles) for 15 days and body weight was evaluated. Quercetin-treated mice lost around 25% of body weight as compared with untreated mice (black squares). ^*∗*^ *P* < 0.05, control mice (*n* = 11), quercetin-treated mice (*n* = 9). (b) Insulin plasma levels were measured using mouse insulin ELISA. Quercetin-treated mice showed a significant decrease in the plasma levels of insulin as compared with control mice. ^*∗*^ *P* < 0.05. (c) and (d) Quercetin treatment affects metabolic performance and exercise abilities. (c) Weightlifting test of forelimbs muscle strength test and (d) Kondziela's inverted test muscle strength and resistance using all four limbs (d). Quercetin-treated mice performed similarly on both strength tests than control mice (c, d). The score obtained in the strength test was normalized by the body weight. Average values were analyzed by two-sample *t*-test (*P* < 0.05), control mice (*n* = 11), and quercetin-treated mice (*n* = 9). Significant differences (*∗*) were detected between control and quercetin-treated mice. (e) Box chart shows the 25th and 75th percentiles. The whiskers show the 5th and 95th percentiles. Additional values are show in box chart, including the minimum (*∗*), median, mean (*▫*), maximum (*∗*), the 1st and 5th percentiles, and 95th percentiles.

Evidence suggests that quercetin is able to modulate metabolism in mice [57, 58]; to evaluate the metabolic status of quercetin-treated mice, we measured body weight and plasma insulin levels. Mice body weight was measured daily for 15 days, decreasing significantly up to 25% upon the treatment with quercetin at 50 mg/kg as compared with control mice (Figure 1(a)). Along with the weight loss, quercetin-treated mice had significantly lower plasma Insulin levels (0.5 ng/mL ± 0.1) than control ones (2.1 ng/mL ± 0.6) (Figure 1(b)), confirming an altered metabolism. Normal average plasma insulin level in mice is 0.6 ± 0.1 ng/mL [59], and the range of native insulin level could be found between 0.1 and 2.9 ng/mL.

Previous reports of epidemiological and clinical studies in humans showed that treatment with quercetin improved cardiovascular health [58]. To test cardiovascular benefits induced by quercetin, we evaluate if quercetin treatment affects exercise abilities enhancing muscle strength and resistance by the weightlifting capability test for forelimbs muscle strength (Figure 1(c)) and the Kondziela's inverted test for resistance and strength (Figure 1(d)). Our studies showed that quercetin-treated mice performed similarly on both strength tests than control mice.

### 3.2. Quercetin Treatment Affects Iron Metabolism and Erythropoiesis

**Figure 2:**
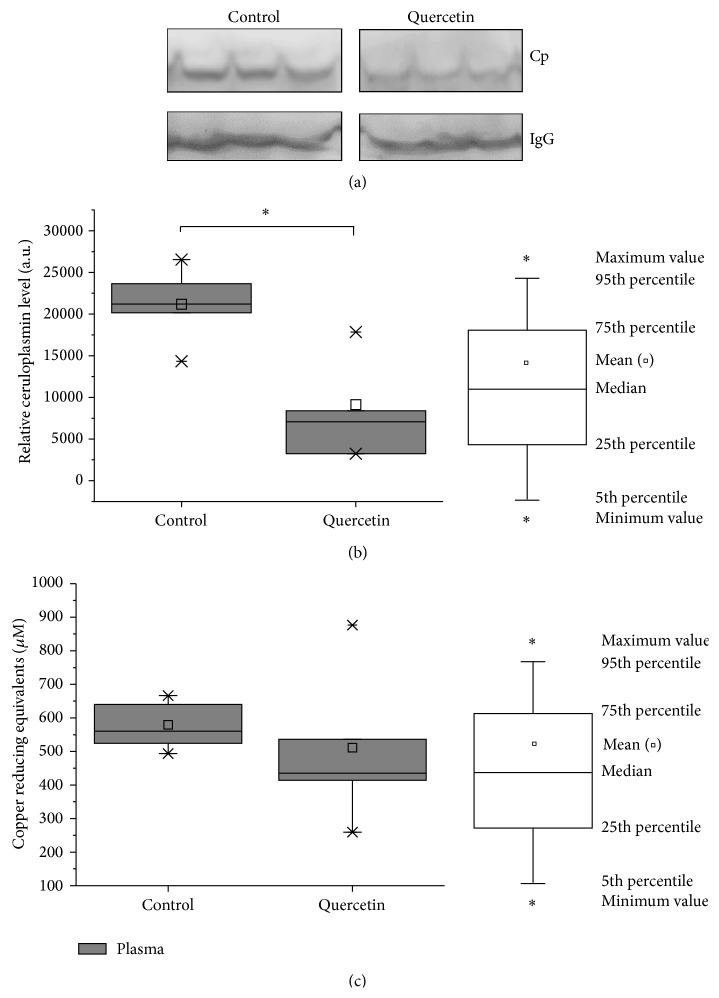
Effect of quercetin treatment on mice iron metabolism. (a) Plasma ferritin levels from quercetin-treated mice were measured by ELISA, as it was described in Section 2. Treatment with quercetin did not affect ferritin levels as compared with untreated mice. (b) Total Antioxidant Capacity was measured in plasma samples from quercetin-treated mice using TAC Oxyselect kit and results are expressed as “*μ*M Copper Reducing Equivalents.” The value of the Copper Reducing Equivalents is directly proportional to the total antioxidant capacity. (c) Mice were treated for 15 days with quercetin and then ceruloplasmin plasma levels were measured by western blot. (d) Densitometry analysis of ceruloplasmin levels performed with the ImageJ software. Ceruloplasmin plasma levels of mice treated with quercetin significant decreased 57%, compared to untreated mice ^*∗*^ *P* < 0.05 compared to control. Control mice, *n* = 11; quercetin-treated mice, *n* = 9. Each bar (box charts) represents the mean ± SD, analyzed by two-sample *t*-test (*P* < 0.05). Significant differences (*∗*) were found between control and quercetin-treated mice. ^*∗*^ *P* < 0.05.

The ferroxidase ceruloplasmin can be used as markers of iron deficiency [60, 61]. Ceruloplasmin is essential for iron homeostasis by favoring cellular iron release [62] and has been described to decrease upon iron deficiency [61–63]. In our study, the levels of ceruloplasmin were significantly reduced in 50% (*P* < 0.05) as compared with control mice (Figures 2(a) and 2(b)). Furthermore, the plasma's Total Antioxidant Capacity (TAC) was evaluated showing a no significant decrease upon the quercetin treatment (Figure 2(c)).

**Figure 3:**
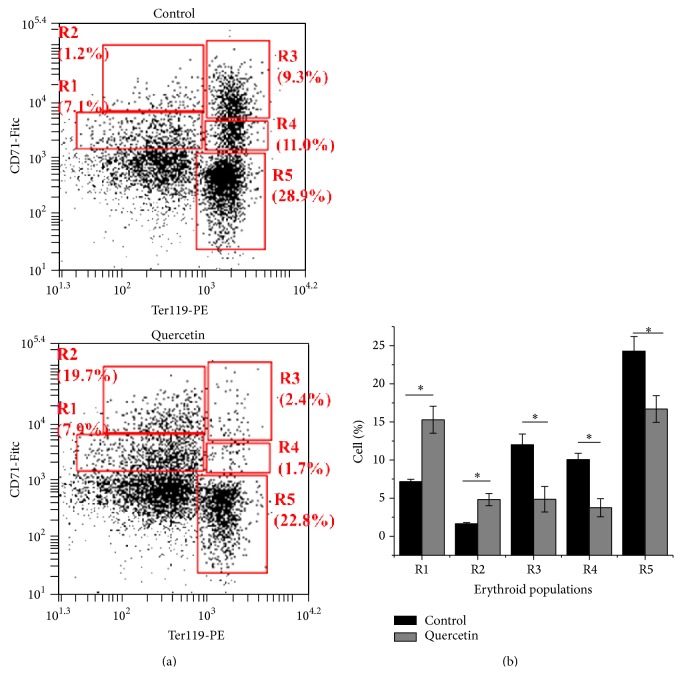
Erythropoietic progression in bone marrow cells is altered in quercetin-treated mice. (a) FACS plots. (a) Five erythroid populations from immature to mature (R1, R2, R3, R4, and R5) are distinguished by CD71 and TER119 expression levels in freshly isolated bone marrow through flow cytometry (CD71^med^-TER119^low^, CD71^high^-TER119^low^, CD71^high^-TER119^high^, CD71^med^-TER119^high^, and CD71^low^-TER119^high^). (b) Quantitative analysis of cell progression. CTR mice, *n* = 11 (red bars); quercetin-treated mice, *n* = 9 (green bars). Each bar represents the mean ± SD, analyzed by two-sample *t*-test (*P* < 0.05). Significant differences (*∗*) were found between control and Quercetin-treated mice. ^*∗*^ *P* < 0.05 compared to control.

The erythropoiesis process is highly dependent on iron transport and metabolism. Flow cytometry on bone marrow isolated cells from quercetin-treated mice displayed a significant delay on erythropoiesis progression as compared with control mice. Immature erythroid populations R1 (CD71^med^-TER119^low^) and R2 (CD71^high^-TER119^low^) showed a significant increase as compared with vehicle-treated mice (Figures 3(a) and 3(b)). On the other hand, the iron dependent cell populations of erythropoiesis for heme and hemoglobin biosynthesis, R3 (CD71^high^-TER119^high^), R4 (CD71^med^-TER119^high^), and R5 (CD71^low^-TER119^high^) were all of them significantly decreased in quercetin-treated mice (Figures 3(a) and 3(b)). These observations suggest that quercetin treatment may induce anemia given the significant delay on erythropoiesis progression (Figures 2 and 3).

### 3.3. Effect of Quercetin on Heart Mitochondrial Function

**Figure 4:**
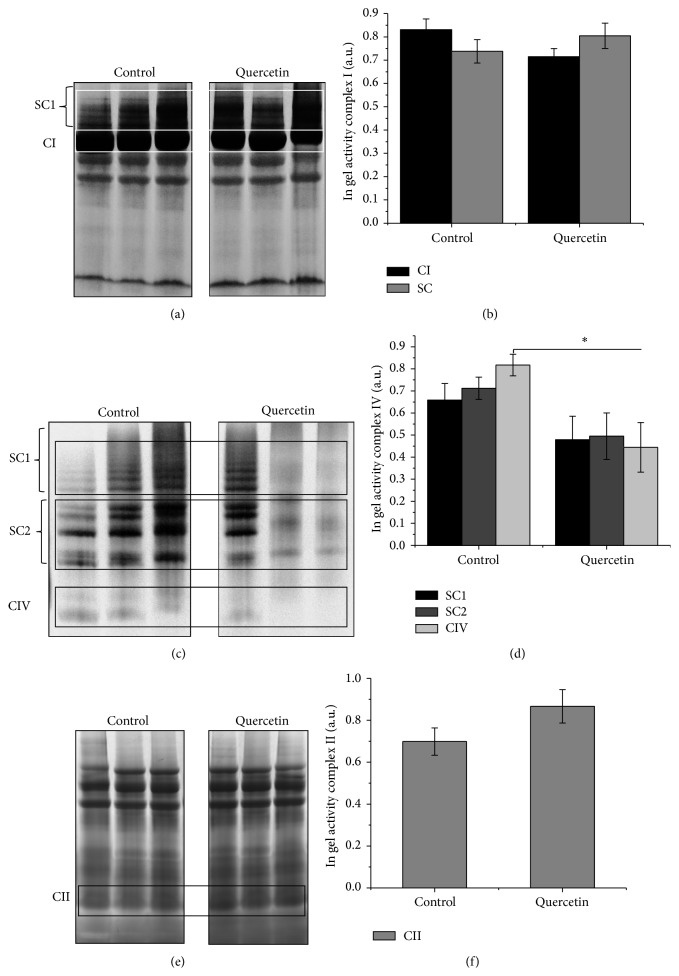
Quercetin treatment affects complexes OXPHOS properties in mice isolated mitochondria. Heart mitochondria isolated from quercetin-treated mice were digitonin solubilized and fractionated by BN-PAGE and then followed by Complex I (a), Complex II (b), and Complex IV (c)* in-gel* activity assays. (a) IGA Complex I densitometry analysis for supercomplex and monomer fraction. (b) IGA Complex IV densitometry analysis for supercomplex and monomer fraction. Images show three independent experiments. Each bar represents the mean ± SD, analyzed by two-sample *t*-test (*P* < 0.05). Abbreviations used are as follows: blue native polyacrylamide gel electrophoresis (BN-PAGE), Complex I (CI), Complex IV (CIV), in-gel activity assay (IGA), and supercomplexes (SC). SC1:CI:CIII_2_:CIV_1−4_; SC2:CIII_2_:CIV_1−4_.

Previous work showed the prooxidant effect of quercetin* in vitro* in the HCT116 human colon tumor cells and liver tissue [15, 17]. In addition, heart function is highly dependent on aerobic metabolism and then mitochondrial function, that is, the oxidative phosphorylation system (OXPHOS) for energy generation [25, 26]. We analyzed the effect quercetin on heart mitochondrial function analyzing respiratory complexes activity in response to quercetin treatment.* In-gel* activity (IGA) was tested for mitochondrial Complexes I, II, and IV as well as for supercomplex rearrangements, using heart mitochondrial proteins from quercetin- and vehicle-treated mice. For Complex I, no significant changes were observed at the level of monomeric Complex I, nor supercomplex rearrangement (Figures 4(a) and 4(b)). Similar results were obtained for Complex II (Figures 4(e) and 4(f)). However, at the level of Complex IV (Figures 4(c) and 4(d)), quercetin treatment significantly reduced the activity of monomeric Complex IV (not being part of the supercomplexes CI:CIII_2_:CIV_1−4_). Altogether, these observations indicate that quercetin significantly altered mitochondrial function through deregulation of Complex IV activity.

**Figure 5:**
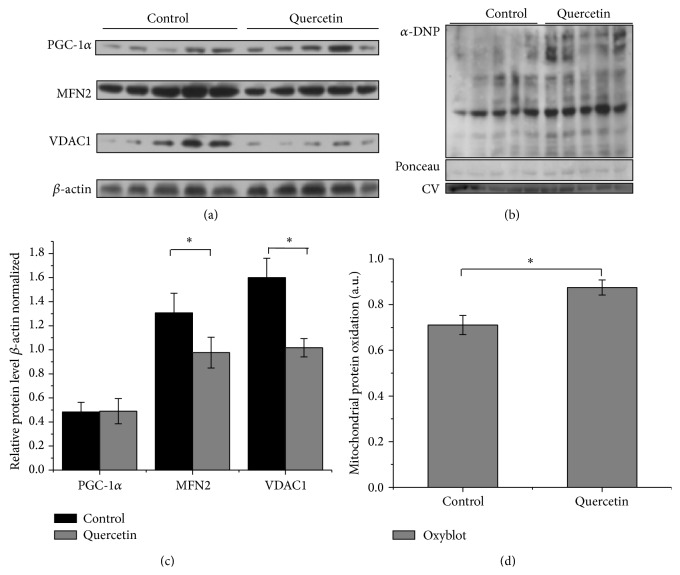
Effect of quercetin treatment on the expression of PGC-1*α*, Mitofusin 2, VDAC, and protein oxidation. (a) Detection of carbonyl groups was performed with the OxyBlot Protein Oxidation Detection Kit. (c) Densitometry quantification of carbonyl groups was made with the ImageJ software. Carbonylation of proteins was normalized by Ponceau staining and Complex V (CV) expression. (b) Expression of mitochondrial proteins. Protein expression of MFN2, PGC-1*α*, and VDAC1 was analyzed in heart isolated mitochondria from control and quercetin-treated mice. *β*-actin was used as a loading control. (d) Densitometry analysis. MFN2, PGC-1*α*, and VDAC1 expressions were normalized by *β*-actin expression. Each bar represents the mean ± SD, analyzed by two-sample *t*-test (*P* < 0.05). Control mice, *n* = 11; quercetin-treated mice, *n* = 9. Each bar represents the mean ± SD, analyzed by two-sample *t*-test (*P* < 0.05). Significant differences (*∗*) were found between control and quercetin-treated mice. *P* < 0.05.

The effect of quercetin on heart mitochondrial function was also studied at the level of mitochondrial biogenesis and mitochondrial dynamics. Protein expression for PGC-1*α*, MFN2, and VDAC was analyzed by immunoblots (Figure 5). PGC-1*α*, the master regulator of mitochondrial biogenesis is upregulated upon energy expenditure and demand [39]. Mitochondrial dynamics (MtDy), given by the balance between fusion and fission events, control not only mitochondrial morphology but rather mitochondrial function, mitochondrial turnover, and bioenergetics. MFN2, a mitochondrial fusion protein located on the outer mitochondrial membrane, has been shown to be upregulated upon stressful conditions [36, 37]. The Voltage-Dependent Anion Carrier (VDAC) also performs several key functions, including regulating the shape and structure of mitochondria, interaction with hexokinase, and apoptosis signaling [64]. The immunoblot results showed quercetin treatment did not affect the expression of PGC-1*α*. On the other hand, VDAC and MFN2 protein expression levels were significantly decreased in quercetin-treated mice (Figures 5(a) and 5(c)). Mitochondrial dysfunction is normally correlated with an increase in the reactive oxygen species (ROS). To evaluate redox status in heart mitochondria, protein oxidation was assessed by the OxyBlot methodology [65]. Treatment with quercetin showed a significant increase in mitochondrial protein oxidation as compared with control (Figures 5(b) and 5(d)).

Altogether, our results suggest that* in vivo* quercetin treatment is associated with a severe mitochondrial dysfunction drastically affecting erythropoiesis and heart mitochondria.

## 4. Discussion

*In vitro* and* in vivo* studies showed that quercetin may exert dual antioxidant and prooxidant properties that depend on tissue and cellular redox state [11–14]. This study shows that quercetin clearly affects heart mitochondrial function and erythropoiesis in mice. We observed the prooxidant capacity of this polyphenolic flavonoid which induces higher levels of carbonylated (oxidized) heart mitochondrial proteins when injected in mice (Figures 5(a) and 5(c)). Associated with this prooxidant property, quercetin treatment induced a significant decrease in the activity of the monomeric Complex IV (Figures 4(e) and 4(f)). In addition, quercetin treatment clearly decreased MFN2 and VDAC levels (Figures 5(b) and 5(d)). All these results are in agreement with the effects of quercetin on mitochondria previously observed* in vitro* [15, 16]. Previous evidence suggests that quercetin interacts with the mitochondrial inner membrane, inducing an inhibition of respiratory chain and decreasing ATP levels [15]. This is relevant because quercetin enters into cytosol and may also reach the mitochondria [4, 66]. Flavonoids can also induce apoptosis in association with prooxidant activities inducing the mitochondrial transition pore (mPTP) [67, 68]. Quercetin induces the opening of mPTP mediated by Fe and Cu [17], to release Ca^2+^ accumulated in mitochondria [15].

Larocca et al. reported in bone marrow from humans adult patients suffering acute leukemia that quercetin could inhibit leukemic cell growth without suppressing normal hematopoiesis [69]. In that study, bone marrow isolated from the patients (*in vitro* experiments) was treated with quercetin every two days during two weeks and the ability of human CD34+ cells to form both BFU-E and CFU-GM was not affected by quercetin, but the quercetin concentration was just 2 × 10^−5^ M (approximately 6 mg/kg) [69]. Bakheet reported that quercetin was not cytotoxic (DNA strand breaks) to bone marrow at the tested doses of 50 mg/kg and 100 mg/kg. In addition, bone marrow ROS production, lipid peroxidation, and GSH/GSSG ratio (reduced and oxidized glutathione) did not show significant variation after treatment of mice [70]. However, these experiments were made for a short time, two days. The effect that we observed on the bone marrow from quercetin-treated mice (*in vivo* experiments) was an evident and significant delay on the erythropoiesis progression (Figure 3), which suggests the development of anemia. In the present study a decreased activity of Complex IV in mice treated with quercetin is clear. This is well correlated with decreased levels of ceruloplasmin (Figure 2). Our previous research [30] exposed that mild copper deprivation in mice is correlated with a decreased protein expression and activity of Complex IV at the level of OXPHOS supercomplexes along with a decrease in the ceruloplasmin levels. Our present results are suggesting a potential interaction between quercetin and copper ions, which is in accordance with* in vitro* observations made by Pękal et al. [71]. Laccases are multicopper oxidases structurally and functionally similar to ceruloplasmin. Laccases and ceruloplasmin exhibit reactivity in the hemoglobin-flavonoid system. Then, quercetin as a flavonoid in the presence of ceruloplasmin may oxidize hemoglobin affecting the viability of red blood cells [72]. Galati et al. reported that the generated phenoxyl radicals from flavonoid oxidation are responsible for oxidation of oxy-hemoglobin directly in red cells and lysis of red cells. It is known that hemoglobin is prone to oxidative damage and unable to transport molecular oxygen [73].

Quercetin has been proposed to increase mitochondrial biogenesis through the regulation of PGC-1*α* pathway. Davis et al. treated mice (and HepG2 cells) with quercetin at 12.5 mg/kg and 25 mg/kg for one week and showed that quercetin increases brain and muscle mitochondrial biogenesis through the activation of PGC-1*α* and sirtuin 1, increasing the levels of mtDNA and cytochrome c in HepG2 cells and mice [74]. On the other hand, Casuso et al. supplemented rats by oral gavage with 25 mg/kg of quercetin combined with exercise during 6-week [13] observing that quercetin supplementation during exercise compromises both the exercise and the quercetin effects on brain mitochondrial content by disrupting the SIRT1-PGC-1*α* pathway. Quercetin impedes exercise-induced adaptations in the brain. Quercetin induced oxidative damage which, in the sedentary condition, is counteracted by modulating antioxidant activity [13]. In this work, we observed that quercetin treatment for two weeks, at 50 mg/kg, did not affect the expression of PGC-1*α* in heart. In contrast, VDAC and MFN2 protein levels showed a significant decrease in quercetin-treated mice (Figures 5(b) and 5(d)). These results suggest that quercetin affects mitochondrial dynamics compromising metabolism, substrate oxidation, and the oxidative phosphorylation system [38]. Alterations in the expression of MFN-2 have been reported to cause a parallel change in protein expression levels of Complexes I, III, and IV [34]. These effects could be related with our observations reported here, where quercetin affects Complex IV activity.

Quercetin-treated mice displayed a weight loss and lower insulin levels, which confirmed an altered metabolism (Figure 1). Vessal et al. show an antidiabetic effect of quercetin in diabetic rats [75]. Li et al. show a decrease in insulin levels and weight loss in fructose-induced hyperinsulinemia [57]. Xia et al. proposed that under a nutritional balanced situation quercetin exerts prooxidant effects, affecting cognition [12]. Thus, depending on the diet, quercetin might have protective or detrimental effects on cell physiology.

## 5. Conclusion

Quercetin is widely used as a dietary supplement in healthy people as an antioxidant. However, our results suggest that quercetin intake affects mitochondrial function in cardiac tissue and also erythropoiesis, which is a warning about the nutraceutical use of this compound.
